# Effects of dexmedetomidine on porcine pulmonary artery vascular smooth muscle

**DOI:** 10.1186/s12871-019-0843-2

**Published:** 2019-09-12

**Authors:** Mami Chikuda, Kenichi Sato

**Affiliations:** Division of Dental Anesthesiology, Department of Reconstructive Oral and Maxillofacial Surgery, School of Dentistry, Iwate Medical University, 1-3-27 Chuo-dori, Morioka, Iwate, 020-8505 Japan

**Keywords:** Porcine pulmonary artery, Dexmedetomidine, Adrenaline, Isometric tension

## Abstract

**Background:**

The α_2_-receptor agonist dexmedetomidine (Dex) has been shown to produce sedative and analgesic effects not only with systemic administration but also when administered in the extradural space and around peripheral nerves. The effects and mechanism of action of Dex on pulmonary arteries, however, have not been determined. This study therefore aimed to investigate the effect of Dex on pulmonary arterial vascular smooth muscle by evaluating changes in isometric contraction tension. We then attempted to determine the effects of Dex on depolarization stimulation and receptor stimulation.

**Methods:**

Endothelium-denuded porcine pulmonary arteries were sliced into 2- to 3-mm rings. We then exposed them to certain substances at various concentrations under different conditions of baseline stimulation (with KCl, adrenaline, caffeine, or histamine) and to α_2_-receptor stimulants or antagonists, or α_1_-receptor antagonists (imidazoline, yohimbine, rauwolscine, prazosin), and different conditions of Ca^2+^ depletion of the intracellular reservoir or extracellular stores. We measured the changes in isometric contraction tension with each addition or change in conditions.

**Results:**

Dex enhanced the contraction induced by high-concentration KCl stimulation. Dex-induced enhancement of contraction induced by high-concentration KCl was completely suppressed by yohimbine and rauwolscine, which are α_2_-receptor antagonists, but not by prazosin. Dex, imidazoline, yohimbine, and rauwolscine reduced the increases in contraction tension induced by the receptor stimulant adrenaline. Dex suppressed the adrenaline-induced increases in contraction tension after depletion of the Ca^2+^ reservoir. In the absence of extracellular Ca^2+^, Dex suppressed the adrenaline- and histamine-induced increases in contraction tension but did not affect caffeine-induced increases.

**Conclusions:**

Dex-enhanced, high-concentration KCl-induced contraction was mediated by α_2_-receptors. Adrenaline-induced contraction was suppressed by the α_2_-receptor stimulant Dex and α_2_-receptor antagonists yohimbine and rauwolscine, suggesting that the effect of Dex on adrenaline-induced contraction is attributable to its α_2_-receptor-blocking action. Dex inhibited receptor-activated Ca^2+^ channels and phosphatidylinositol-1,4,5-triphosphate-induced Ca^2+^ release but not Ca^2+^-induced Ca^2+^ release.

## Background

The α_2_-receptor agonists have been shown to produce sedative and analgesic effects not only with systemic administration but also when administered in the extradural space and around peripheral nerves [1–3]. Among the α_2_-receptor agonists, dexmedetomidine hydrochloride (Dex) is added to local anesthetics to increase their potency and extend their duration of action. For example, administration of Dex 0.5 μg/kg with 0.5% lidocaine to the brachial plexus for brachial nerve block significantly extends the duration of the local anesthetic’s effect [2] and provides postoperative analgesia with a single administration. In dentistry, the current use of local anesthetics containing adrenaline may cause abnormal blood pressure increases, leading to adverse effects such as cerebrovascular disease. To prevent these complications, the adrenaline in local anesthetics should be replaced with an additive that causes smaller fluctuations in the circulation during local anesthesia [4–6]. The use of Dex-containing lidocaine as a local anesthetic may offer simultaneous prolongation and potentiation of anesthetic effects and may be useful for dental treatment in patients with cardiovascular disease. Dex has thus attracted attention and is being tested in clinical studies as an additive agent for dental local anesthesia. The effects and mechanism of action of Dex on the cardiopulmonary vascular system should therefore be clarified. Although several in vivo studies have shown the effects of Dex on aortic or coronary arteries in various animals, there are few reports regarding its effect on other peripheral vessels. The pulmonary artery has been relatively unexplored, even though it is a prominent artery that nourishes the lungs. Pulmonary vasoconstriction, with its resultant progressive elevation of pulmonary arterial resistance and pressure, plays a central role in pulmonary arterial hypertension, which could be fatal [7, 8]. Nevertheless, there are only a few reports of the effects of Dex on the pulmonary artery. The diversity of effects of Dex on smooth muscle precludes guessing its effects on any individual smooth muscle.

To elucidate the effects and mechanism of action of Dex on vascular smooth muscle of the pulmonary artery, we measured isometric contraction tension in the artery. We then attempted to determine the effects of Dex on depolarization stimulation and receptor stimulation. To evaluate Dex’s possible mechanisms of action, we investigated components of the two most important pathways involved in intracellular Ca^2+^ fluctuations during vascular smooth muscle contraction—i.e., intracellular influx of extracellular Ca^2+^ and release of stored intracellular Ca^2+^ within the cell.

## Methods

This study was approved by the Institutional Review Committee on the Ethics of Animal Experiments of Iwate Medical University. All experiments were conducted in accordance with the Institutional Animal Care and Use Committee guidelines (Ethical number 26–010).

### Reagents and solutions

All chemicals were obtained from Wako Pure Chemical Industries (Osaka, Japan).

In all experiments, air-equilibrated Hank’s balanced salt solution (HBSS) was used to maintain the arteries under the resting condition. HBSS was composed of 137 mM NaCl, 5.4 mM KCl, 0.8 mM MgSO_4_, 1.26 mM CaCl_2_, 0.34 mM Na_2_HPO_4_, 0.44 mM KH_2_PO_4_, 4.2 mM NaHCO_3_, and 5.55 mM glucose (pH 7.34). All other salt solutions used as perfusates were prepared by modifying the HBSS. Isotonic 60 mM KCl solution was prepared by replacing the NaCl in the HBSS solution with an equimolar amount of KCl.

### Arterial ring preparation and isometric tension measurement

The pigs were killed as part of a routine procedure in the slaughterhouse where we obtained the porcine lungs. The one-third of the pulmonary arteries that were 2–3 mm in diameter were excised from the lung of a 6-month-old slaughtered pig and cut into rings 2–3 mm in length. The endothelium, which was rubbed gently against the thin arm of stainless steel tweezers [8, 9], was then denuded and the rings inverted (inner surface facing outward) to prepare specimens of pulmonary arterial vascular smooth muscle. It was confirmed that 3 μM acetylcholine induced relaxation of the arterial rings, which disappeared after this procedure. The arterial rings were maintained in HBSS at 5 °C until used for the assessments.

Specimens were placed in a perfusion chamber (with 3 ml of perfusate), and a resting tension of 4–7 mN was applied. The perfusate, adjusted to 37 °C, flowed at a rate of 1.6 ml/min controlled by a peristaltic pump (SMP-23; Tokyo Rikakikai Co., Fujisawa, Japan). After perfusion with HBSS for 30 min, various stimulants were administered, and the resulting contraction tension after each addition was measured via the following steps. The end of the specimen in the perfusion chamber was fixed to a manipulator (M-152; Narishige, Tokyo, Japan) and the other end to a tension transducer (UL-2GR; Minebea, Tokyo, Japan) using a tungsten wire. Data were recorded on PowerLab® (ADInstruments, Bella Vista, Australia) via a pressure amplification unit (N4438; NEC San-ei, Tokyo, Japan).

The experiments were undertaken, as follows.

**Fig. 1:**
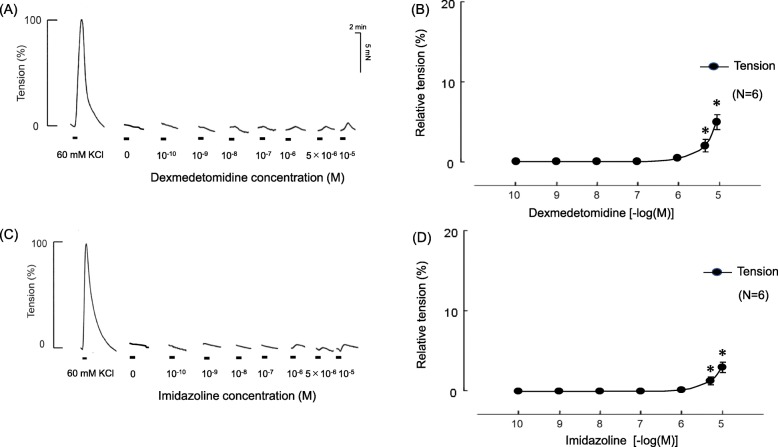
Direct effects of various concentrations of dexmedetomidine (Dex) and imidazoline on pulmonary arterial vascular smooth muscle. Representative traces show the effects of Dex (**a**) and imidazoline (**b**) on contraction tension. The tensions were measured relative to the KCl baseline. Statistical analysis was performed with one-way analysis of variance followed by Dunnett’s multiple comparison procedure. **c** and **d** Analyzed data. Six arterial samples were tested under each condition. *P < 0.05, compared with 0 M Dex

**Fig. 2:**
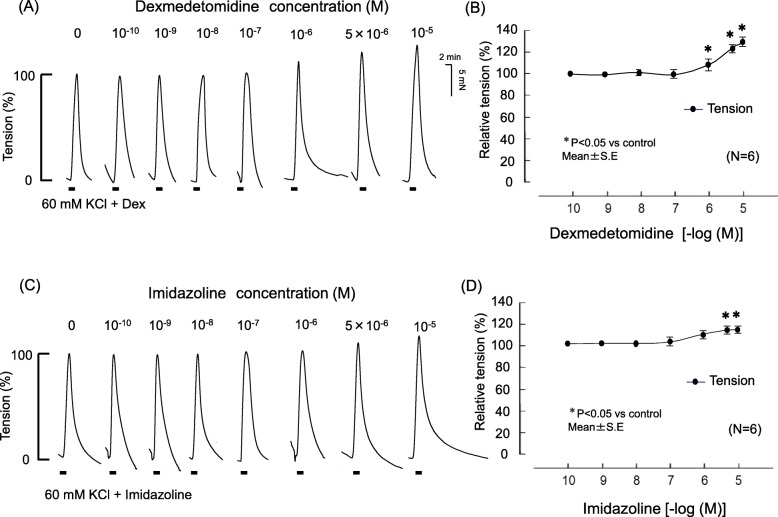
Effects of dexmedetomidine and imidazoline on contraction tension with 60 mM KCl. Representative traces show the effects of dexmedetomidine and imidazoline on contraction tension in endothelium-denuded porcine pulmonary artery (**a**, **c**) induced with 60 mM KCl. **b** and **d** Analyzed data. The contraction tensions were normalized to 60 mM KCl in each set and expressed as a relative value (in percents). The tensions were measured relative to the KCl baseline. Statistical analysis was performed with one-way analysis of variance followed by Dunnett’s multiple comparison procedure. Each bar and accompanying line indicate the mean and SE of the group. Six samples were included in each group. *P < 0.05, compared with 60 mM KCl

1. At the beginning of each experiment, after approximately 2 min of perfusion with 60 mM KCl solution (Figs. 1, 2, 4, 6 and 7) or 5 μM adrenaline (Fig. 5), the contraction tension was measured, recorded, and used as the control value for that experiment.
2. The concentration–response relations for Dex and imidazoline were determined by adding each to HBSS or 60 mM KCl (Figs. 1 and 2). The contraction tension measured in 60 mM KCl at the beginning of the experiment was used as the control value. The concentration–response relations for Dex, imidazoline, yohimbine, and rauwolscine were determined by adding each to 5 μM adrenaline (Fig. 5) for 2 min and then monitoring the reaction for almost 20 min. The contraction tension measured in 5 μM of adrenaline at the beginning of the experiment was used as the control value.
3. After recording the amplitude of the control contraction induced by KCl, 5 μM Dex was added to 60 mM KCl and the response recorded. Then, 1 μM of yohinbine, rauwolscine, and prazosin each were added to 60 mM KCl containing 5 μM Dex (Fig. 4) for 2 min and then monitored for almost 20 min to determine the responses. The contraction tension measured in 60 mM KCl at the beginning of the experiment was used as the control value.
4. After depleting the intracellular Ca^2+^, 5 μM Dex was added to 5 μM adrenaline and the response recorded (Fig. 6). The contraction tension measured in 60 mM KCl at the beginning of the experiment was used as the control value.
5. After depletion of extracellular Ca^2+^, the effect of 5 μM Dex in Ca^2+^-free HBBS, was determined by adding 5 μM Dex to 5 μM adrenaline, 5 μM histamine, and 25 mM caffeine, respectively, for 2 min (Fig. 7). Each solution was then monitored for almost 20 min to determine the responses. The contraction tension measured in 60 mM KCl at the beginning of the experiment was used as the control value.

The preliminary experiments showed that, after application of each of these substances, an almost 20-min interval was sufficient for the tension to return to the control level (Figs. 1, 2, 4, 5, 6 and 7). For all experiments, each new set of ingredients was carried out with fresh preparations because it has been suggested that down-regulation of α_2_-adenoreceptors often occurs after prolonged α_2_-agonist treatment [10, 11].

### Statistical analysis

Values are presented as means ± SEM. Statistical analysis was performed using SPSS for Windows 10 (IBM SPSS Statistics 26; IBM Corp., Armonk, NY, USA). Differences between the means of two groups were evaluated using Student’s t test. Differences among multiple groups were evaluated with one-way analysis of variance followed by Dunnett’s or Scheffe’s multiple comparison procedure. Differences were considered significant at *p* < 0.05.

## Results

### Direct effects of various concentrations of Dex and imidazoline on pulmonary arterial vascular smooth muscle

Significant changes in contraction tension were observed with the addition of Dex and imidazoline at concentrations of ≥5 μM (Fig. 1).

### Effects of various concentrations of Dex and imidazoline on high-concentration KCl-induced contraction tension

Dex enhanced the contraction induced by high KCl stimulation, with the increases reaching significance at Dex concentrations of ≥1 μM and imidazoline concentrations of ≥5 μM (Fig. 2).

### Comparison between the percentages of changes in direct effects of Dex or imidazoline on pulmonary arterial vascular smooth muscle and that of changes in effects of Dex or imidazoline on contraction tension induced with 60 mM KCl

**Fig. 3:**
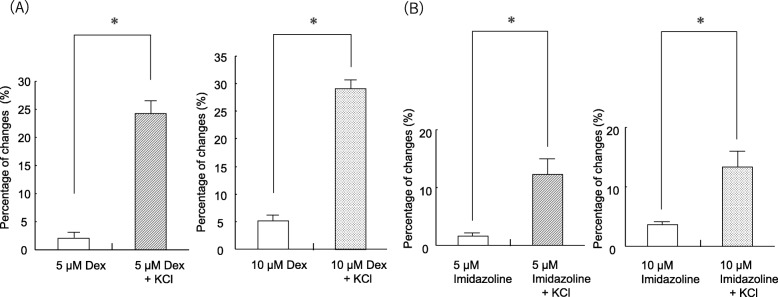
Comparison between the percent changes in direct effects of dexmedetomidine (Dex) on pulmonary arterial vascular smooth muscle (Fig. 1) and that of changes in the effects of Dex on contraction tension induced with 60 mM KCl (Fig. 2). The percent change in Dex on contraction tension induced with 60 mM KCl was measured relative to the contraction tension baseline induced with KCl. Each bar and accompanying line indicate the mean and SE of a group. Statistical analysis was performed with Student’s t test. *P < 0.05, compared with the percent changes in direct effects of Dex

There were significant differences between the amount of changes in direct effects of Dex and imidazoline on pulmonary arterial vascular smooth muscle and that of changes in effects on contraction tension induced with 60 mM KCl (Fig. 3).

### Effects of yohimbine, rauwolscine, and prazosin on high-concentration KCl-induced contraction tension

**Fig. 4:**
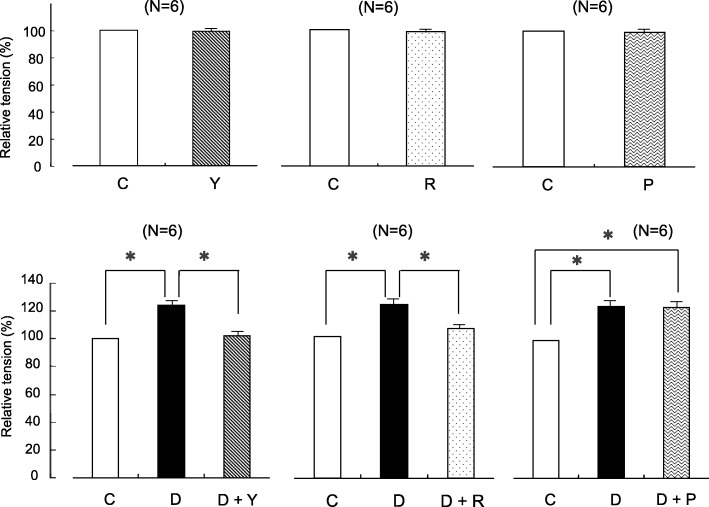
Effects of yohimbine, rauwolscine, and prazosin on contraction tension induced with 60 mM KCl and with dexmedetomidine (Dex)-enhanced responses to 60 mM KCl. Each drug was used at a concentration of 5 μM. Changes in contraction tension were normalized to 60 mM KCl in each set and expressed as a relative value (in percents). Statistical analysis was performed with Student’s t test between the means of two groups and one-way analysis of variance followed by Scheffe’s multiple comparison procedure among the three groups. Each bar and accompanying line indicate the mean and SE of a group. Six samples were included per group. *P < 0.05, compared with 60 mM KCl or Dex. C, control; Y, yohimbine; R, rauwolscine; P, prazosin; D, dexmedetomidine

Yohimbine, rauwolscine, and prazosin had no significant effect on 60 mM KCl-induced contraction tension in pulmonary arterial vascular smooth muscle. Increases in contraction tension by stimulation with 60 mM KCl containing Dex were significantly suppressed by yohimbine and rauwolscine, which are α_2_-receptor antagonists, although there was no significant difference observed with prazocin, an α_1_-receptor antagonist (Fig. 4).

### Effects of various concentrations of Dex, imidazoline, yohimbine, and rauwolscine on adrenaline-induced contraction tension

**Fig. 5:**
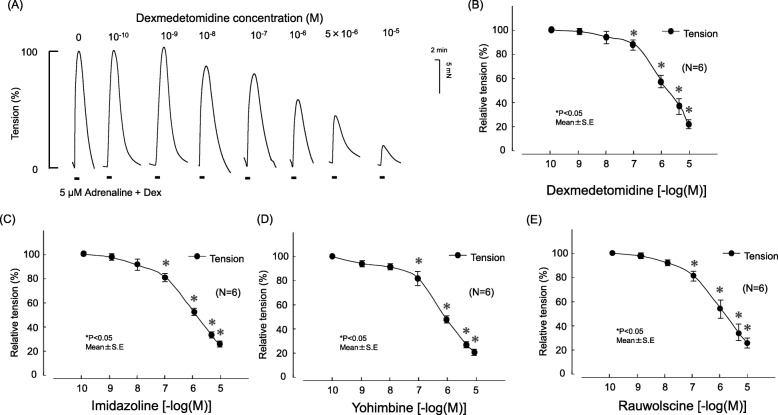
Effects of dexmedetomidine (Dex), imidazoline, yohimbine, and rauwolscine on contraction tension induced with 5 μM adrenaline. **a** Representative traces show the effects of Dex on tension. **b**–**e** Analyzed data. Changes in contraction tension were normalized to 5 μM adrenaline in each set and expressed as a relative value (in percents). The tensions were measured relative to the adrenaline baseline. Statistical analysis was performed with one-way analysis of variance followed by Dunnett’s multiple comparison procedure. Each bar and accompanying line indicate the mean and SE of a group. There were six samples per group. *P < 0.05, compared with 5 μM adrenaline

Dex, imidazoline, yohimbine, and rauwolscine each decreased adrenaline-induced increases in contraction tension in a concentration-dependent manner in pulmonary arterial vascular smooth muscle (Fig. 5). The 50% inhibitory concentrations were 2.083 μM for Dex, 0.8996 μM for imidazoline, 0.376 μM for yohimbine, and 0.5702 μM for rauwolscine.

### Effects of Dex on adrenaline-induced contraction tension with Ca^2+^ reservoir depletion

**Fig. 6:**
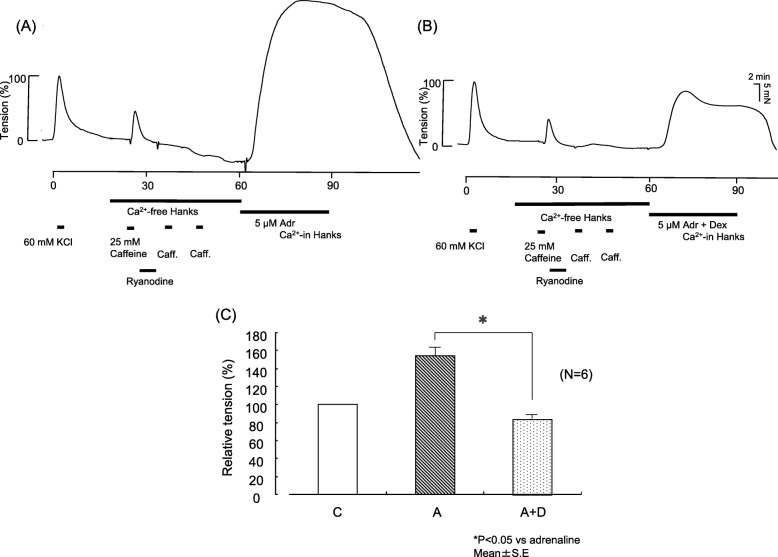
Effects of dexmedetomidine (Dex) on contraction tension induced with 5 μM adrenaline in Ca^2+^-containing Hanks balanced salt solution (Hanks) after depletion of the intracellular Ca^2+^ stores and extracellular Ca^2+^. Representative traces show the effects of Dex on contraction tension in endothelium-denuded pulmonary artery (**a**, **b**) induced with 5 μM adrenaline. **c** Analyzed data. After the Ca^2+^ reservoir was depleted, a Ca^2+^-containing Hanks solution containing 5 μM adrenaline or Ca^2+^-containing Hanks solution containing 5 μM adrenaline supplemented with 5 μM Dex was administered for approximately 15 min, as indicated by the thick black bars below the contraction traces. Changes in contraction tension were normalized to 60 mM KCl in each set and expressed as a relative value (in percents). The tensions were measured relative to the KCl baseline. Each bar and accompanying line indicate the mean and SE of a group. Six samples were included in each group. Statistical analysis was performed with Student’s t test. *P < 0.05, compared with adrenaline. C, control; Adr, adrenaline; D, dexmedetomidine; Caff, caffeine

The first administration of caffeine, which caused the intracellular reservoir to release Ca^2+^ during perfusion with Ca^2+^-free HBSS, induced a transient increase in contraction tension. The second and third caffeine doses, which were administered after fixing the Ca^2+^-induced Ca^2+^ release (CICR) channels in the open state with ryanodine, induced no appreciable changes in contraction tension. When Ca^2+^-containing adrenaline was administered in this state, the contraction tension slowly increased and then remained in a steady state (control). The Ca^2+^-containing adrenaline solution also containing Dex administered under the same conditions as the control induced changes in contraction tension similar to those in the control, although the maximum value was significantly lower (Fig. 6).

### Effects of Dex on adrenaline-, caffeine- and histamine-induced contraction tension in the absence of extracellular Ca^2+^

**Fig. 7:**
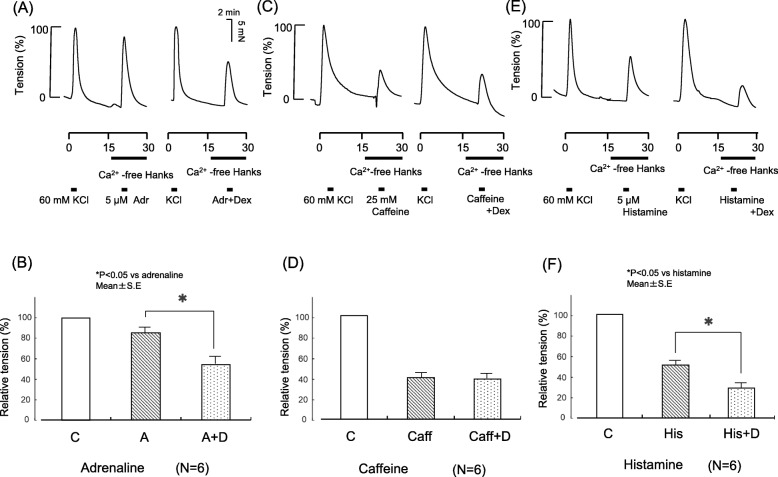
Effects of dexmedetomidine (Dex) on contraction tension induced with 5 μM adrenaline, histamine, and 25 mM caffeine with depletion of extracellular Ca^2+^. **a**, **c**, **e** Representative traces show the effects of Dex on contraction tension. **b**, **d**, **f** Analyzed data. Changes in contraction tension were normalized to 60 mM KCl in each set of experiments and expressed as a relative value (in percents). The tensions were measured relative to the KCl baseline. Each bar and accompanying line indicate the mean and SE of a group. Six samples were included in each group. Statistical analysis was performed with Student’s t test. *P < 0.05, compared with adrenaline, histamine. C, control; A, adrenaline; D, dexmedetomidine; Caff, caffeine; His; histamine

When Ca^2+^ was present in the intracellular reservoir but absent in the extracellular fluid, contraction tension transiently increased with administration of adrenaline, caffeine, and histamine and then rapidly decreased (control). Dex-containing adrenaline, caffeine, and histamine administered under the same conditions as the control induced changes in contraction tension similar to those in the control. Dex-containing adrenaline and histamine produced significantly lower maximum values, whereas Dex-containing caffeine had no effect on contraction tension (Fig. 7).

## Discussion

This study produced two major findings. First, in porcine pulmonary arterial vascular smooth muscle, Dex increased contraction tension that had been induced by depolarization stimulation with high-concentration KCl and reduced the increases in contraction tension induced by adrenaline receptor stimulation. These effects were concentration-dependent in both cases. Second, Dex suppressed receptor-activated Ca^2+^ channels (RACCs), which allow extracellular Ca^2+^ into the cells, and phosphatidylinositol-1,4,5-triphosphate (IP_3_)-induced Ca^2+^ release (IICR), which releases intracellular Ca^2+^. Dex did not suppress CICR.

In porcine pulmonary arterial vascular smooth muscle, Dex enhanced the contraction induced by high-concentration KCl stimulation (Figs. 2 and 3). Conversely, the Dex-induced enhancement of contraction induced by high-concentration KCl was completely suppressed by yohimbine and rauwolscine (α_2_-receptor antagonists) but not by prazosin (α_1_-receptor antagonist) (Fig. 4). These results suggest that Dex’s enhancing effect on vascular smooth muscle contraction induced by high-concentration KCl depolarization is mediated by an α_2_-receptor mechanism.

A study of endothelium-denuded human gastroepiploic arteries showed that the enhancement of high-concentration KCl-induced vascular smooth muscle contraction induced by adding Dex was completely antagonized by the α_2_-receptor antagonists yohimbine and rauwolscine, leading the authors to conclude that the enhancing effect of Dex is mediated by α_2_ receptors [11]. Another study, on the human forearm, showed that the vasoconstriction effect of Dex after administration of a β- or α_2_-receptor antagonist was completely antagonized by the α_2_-receptor antagonist yohimbine [12].

In general, high-concentration KCl-induced contraction of vascular smooth muscle is mediated by increased Ca^2+^ brought about by an influx of extracellular Ca^2+^ via voltage-dependent Ca^2+^ channels (VDCCs). These channels open in response to cell membrane depolarization, resulting in intracellular CICR via ryanodine receptors on endoplasmic reticulum (ER) [13]. Dex-induced increases in high-concentration KCl-induced contraction tension may promote VDCC-mediated influx of extracellular Ca^2+^ and/or CICR. Stimulation with caffeine activates ryanodine receptors on the ER and promotes CICR to increase Ca^2+^, resulting in contraction. In the present experiment, Dex had no effect on caffeine-induced increases during contraction tension in the Ca^2+^-free HBBS solution (Fig. 7). Therefore, the mechanism by which Dex increases depolarization-induced contraction of the pulmonary arterial vascular smooth muscle is not facilitation of CICR from the Ca^2+^ reservoir. Rather, the increase is likely to result from facilitated influx of extracellular Ca^2+^ via VDCCs. α_2_-Receptor-induced contraction of human subcutaneous resistance arteries depends, at least in part, on Ca^2+^ influx via L-type VDCCs [14]. α_2_-Receptor stimulants directly promote VDCCs by a mechanism that depends on a G protein associated with protein kinase C activation [15]. It has also been reported that α_2_-receptor stimulation in rat saphenous vein vascular smooth muscle results from depolarization of the cell membrane, which indirectly enhances Ca^2+^-dependent contraction and Ca^2+^ sensitivity through VDCC activation [16]. Because Dex has an imidazole group, it is believed to act not only on the α_2−_receptor but also on imidazoline receptors [17]. We therefore administered imidazoline, which is an imidazoline-receptor stimulant, and compared its effects with those of Dex. Imidazoline also increases contractions resulting from depolarization with high-concentration KCl (Figs. 2, 3).

The α_2_-receptor stimulant Dex, imidazoline-receptor stimulant imidazoline, and α_2_-receptor antagonists yohimbine and rauwolscine produced concentration-dependent decreases in contraction induced by the α_1_α_2_-receptor stimulant adrenaline (Fig. 5). Dex and imidazoline suppressed contraction resulting from adrenaline, suggesting that receptor stimulants containing an imidazoline group inhibit receptor stimulation involving both α_1_ and α_2_. In the present study, adrenaline-induced contraction was suppressed by the α_2_-receptor stimulant Dex, the imidazoline-receptor stimulant imidazoline, and the α_2_-receptor antagonists yohimbine and rauwolscine. These findings suggest that the effect of Dex on adrenaline-induced contraction is attributable to its α_2_-receptor-blocking action.

Dex inhibited RACC and IICR but not CICR. Cell membrane Ca^2+^ channels regulated by receptor stimulation include RACCs, which are receptors that have a channel function coupled with receptor stimulants and that mediate the influx of extracellular Ca^2+^. Receptor stimulants activate phospholipase C by activating G protein-coupled receptors on the cell membrane, resulting in the production of IP_3_ from phosphatidylinositol, a lipid component of the cell membrane. IP_3_ production leads to activation of IICR from the intracellular reservoir [18]. Influx of extracellular Ca^2+^ and IP_3_ activate ryanodine receptors on the ER, causing CICR from the Ca^2+^ reservoir. Both IP_3_ and ryanodine receptors, which are present on the ER, play an important role in the regulation of Ca^2+^ release [19]. Vascular smooth muscle contraction is regulated by changes in the Ca^2+^ sensitivity of contraction proteins through phospholipase C activation by receptor stimulation [20].

The present study showed that Dex reduced the increases in contraction tension induced by the receptor stimulant adrenaline, suggesting that it suppressed RACC-mediated influx of extracellular Ca^2+^, IICR, and/or CICR (Fig. 5). Dex’s suppression of adrenaline-induced increases in contraction tension after depletion of Ca^2+^ suggest that Dex reduces the RACC-mediated influx of extracellular Ca^2+^ (Fig. 6). Dex’s suppression of adrenaline-induced increases in contraction tension in the absence of extracellular Ca^2+^ suggest that Dex suppresses IICR and/or CICR (Fig. 7). In the absence of extracellular Ca^2+^, Dex did not affect caffeine-induced increases in contraction tension (Fig. 7). Caffeine stimulation activates ryanodine receptors on the ER and promotes CICR to induce contraction [13]. This mechanism suggests that Dex suppresses IICR because it did not suppress CICR.

We also conducted experiments with histamine to confirm that Dex suppresses IICR. Receptor stimulation by histamine is coupled with phospholipase C via Gq, a G protein-mediated, seven-transmembrane receptor. Ca^2+^ is recruited via IP_3_ as a second messenger. Contraction then occurs via diacylglycerol-mediated activation of protein kinase C [21]. Thus, histamine is believed to act specifically on IICR [22].

Our previous study showed that receptor stimulation in the absence of Ca^2+^ in the extracellular fluid, and following depletion of the Ca^2+^ reservoir with caffeine and ryanodine, did not cause any changes in contraction tension [18]. This finding indicates that IP_3_ receptor stimulation results in no Ca^2+^ release from the Ca^2+^ reservoir when ryanodine receptors are fixed in the open state. The experiment showed that the histamine-induced increase in contraction tension was reduced in the absence of extracellular Ca^2+^, suggesting that Dex suppresses IICR.

When the 6-month-old pig whose tissues were used in the present experiments was euthanized, the major stress of the animal could have produced depletion of the Ca^2+^ reservoir. All pigs at that location are euthanized routinely with an electrical method. Hence, the euthanasia protocol did not introduce bias in the results.

The selection of Dex at a concentration of 5 μM was based on the following data. Figure 2 shows that Dex increased the 60 mM KCl-induced contraction tension, with the observed increases reaching significance at a Dex concentration of ≥1 μM and with the increases reaching ≥20% at Dex concentrations of ≥5 μM. We accepted that the 50% inhibitory concentration of Dex was 2.083 μM based on a dose-dependent curve (Fig. 5). We therefore needed 60–70% maximum inhibition. Hence, we decided to use 5 μM as the cutoff. Dex at high doses activates the α_2_β-receptors distributed in vascular smooth muscle, causing hypertension resulting from contraction of vascular smooth muscle. At low doses, Dex causes hypotension resulting from vasodilation and bradycardia due to parasympathetic dominance [19]. The blood concentration of Dex required to maintain a sedative effect in humans is reported to be similar, at 1.0 × 10^− 9^ g/mL (i.e., 4.0 × 10^− 9^ mol/L) [23, 24]. The present results show that Dex had no effect on vasoconstrictor responses in porcine pulmonary arteries when applied in clinically effective concentrations. The previously reported systemic effects of Dex observed in the clinical setting, including decreased blood pressure, may not be the result of direct actions on vascular smooth muscle but could be due to decreased central and peripheral sympathetic nervous system activity [11]. One clinical report also suggested that, at large doses (> 10^− 8^ mol/L), Dex increases peripheral vascular resistance, leading to increased blood pressure [13]. Although the mechanism of blood pressure increase is unclear, it cannot be ruled out that the vasoconstrictor effects of Dex shown in this study (i.e., those mediated by VDCC activation or in case of accidental intravenous administration) may be relevant in such cases.

## Conclusions

To elucidate the effects and mechanism of action of Dex on vascular smooth muscle of the pulmonary artery, we measured isometric contraction tension in that artery. Dex increased the contraction tension resulting from depolarization stimulation by high-concentration KCl. The enhancement of high-concentration KCl-induced contraction when adding Dex was completely antagonized by the α_2_-receptor antagonists yohimbine and rauwolscine. Thus, Dex’s enhancing effect was mediated by α_2_-receptors. Adrenaline-induced contraction was suppressed by the α_2_-receptor stimulants Dex and imidazoline and the α_2_-receptor antagonists yohimbine and rauwolscine, suggesting that the effect of Dex on adrenaline-induced contraction is attributable to its α_2_-receptor-blocking action. Dex suppressed the adrenaline-induced increases in contraction tension after depletion of Ca^2+^ reservoir. In the absence of extracellular Ca^2+^, Dex suppressed the adrenaline- and histamine-induced increases but did not affect caffeine-induced increases in contraction tension. Also, Dex inhibited RACC and IICR but not CICR.

## Data Availability

The datasets used and/or analyzed during the current study are available from the corresponding author on reasonable request.

## Abbreviations

CICR: Ca^2+^-induced Ca^2+^ release
Dex: Dexmedetomidine
ER: Endoplasmic reticulum
HBSS: Hank’s balanced salt solution
IICR: IP_3_-induced Ca^2+^ release
IP_3_: Phosphatidylinositol-1,4,5-triphosphate
RACC: Receptor-activated Ca^2+^ channel
VDCC: Voltage-dependent Ca^2+^ channel
